# Self-Reported Childhood Maltreatment and Traumatic Events among Israeli Patients Suffering from Fibromyalgia and Rheumatoid Arthritis

**DOI:** 10.1155/2017/3865249

**Published:** 2017-01-11

**Authors:** Raneen Hellou, Winfried Häuser, Inbal Brenner, Dan Buskila, Giris Jacob, Ori Elkayam, Valerie Aloush, Jacob N. Ablin

**Affiliations:** ^1^Internal Medicine F, Tel Aviv Sourasky Medical Center, Tel Aviv, Israel; ^2^Department Internal Medicine I, Klinikum Saarbrücken, Saarbrücken, Germany; ^3^Department of Psychosomatic Medicine, Technische Universität München, Munich, Germany; ^4^Shalvata Mental Health Center, Hod Hasharon, Israel; ^5^Department of Medicine H, Soroka Medical Center, Ben Gurion University of the Negev, Beer Sheva, Israel; ^6^Faculty of Health Sciences, Ben Gurion University of the Negev, Beer Sheva, Israel; ^7^Institute of Rheumatology, Tel Aviv Sourasky Medical Center, Tel Aviv, Israel; ^8^Sackler School of Medicine, Tel Aviv University, Tel Aviv, Israel

## Abstract

*Objective*. The association between Fibromyalgia Syndrome (FMS) and childhood maltreatment and adversity has frequently been proposed but limited data exists regarding the transcultural nature of this association.* Methods*. 75 Israeli FMS patients and 23 Rheumatoid Arthritis (RA) patients were compared. Childhood maltreatment was assessed by the Childhood Trauma Questionnaire (CTQ) and potential depressive and anxiety disorders were assessed by the Patient Health Questionnaire-4. FMS severity was assessed by the Widespread Pain Index (WPI), the Symptom Severity Score (SSS), and the FIQ. PTSD was diagnosed according to the DSM IV. RA severity was assessed by the RA Disease Activity Index. Health status was assessed by the SF-36.* Results.* Similar to reports in other countries, high levels of self-reported childhood adversity were reported by Israeli FMS patients. PTSD was significantly more common among FMS patients compared with RA patients, as well as childhood emotional abuse and physical and emotional neglect. Levels of depression and anxiety were significantly higher among FMS patients.* Conclusion*. The study demonstrated the cross cultural association between FMS and childhood maltreatment, including neglect, emotional abuse, and PTSD. Significant differences were demonstrated between FMS patients and patients suffering from RA, a model of an inflammatory chronic rheumatic disease.

## 1. Introduction

Fibromyalgia Syndrome (FMS) is a condition characterized by the presence of chronic widespread pain accompanied by chronic fatigue, disturbed sleep, and a spectrum of symptoms associated with amplified pain processing within the central nervous system [1, 2].

Several lines of evidence indicate the central nervous system in the pathogenesis of FMS. Thus, functional neuroimaging has demonstrated increased response of the CNS to pain stimulation among FMS patients [3], as well as altered patterns of resting connectivity [4–6]. Hence, FMS has been described as a prototype of a condition in which central sensitization of pain plays a major role [7]. In epidemiological studies, up to 2.5% of the Israeli population have been found to meet criteria for the classification of FMS [8], a figure similar to those found in other populations [9, 10]. A significant overlap has been described between FMS and a spectrum of psychiatric comorbidities, including anxiety and PTSD [11, 12].

While the etiology of FMS remains incompletely understood, current opinion considers this complex condition to represent the end-result of an interaction between a genetic predisposition [13, 14] and a sequence of life-long triggering events which impact upon the central nervous system. These presumed triggers may include such diverse factors as physical trauma [15, 16], infection [17], physical inactivity and obesity [18], sleep disorders [19], and inflammatory joint disease [20, 21]. Early life traumatic experience has been long implicated as a possible predisposing factor for the development of FMS [22]. The mechanisms through which such events as sexual abuse and other massively stressful events might impact upon the developing nervous system of a child remain incompletely understood but constitute an area of great theoretical interest. Previous research has demonstrated transcultural similarity regarding the role of childhood traumatization on the development of FMS [23] in a study comparing patient populations in Germany and the USA. Similar studies have yet to be conducted among other more diverse populations; moreover, the role of childhood trauma in FMS has not been previously compared with the role it may have on other rheumatic conditions. In the current study, we have attempted to establish the prevalence of childhood trauma and PTSD among Israeli FMS patients, a population with distinct and unique cultural and ethnic characteristics, and to compare these results with a sample of Israeli patients suffering from Rheumatoid Arthritis (RA), a prototypical inflammatory joint disorder. We have also compared our current results with the aforementioned results achieved in the German and American populations.

## 2. Methods

### 2.1. Participants and Settings

All patients were recruited at the Rheumatology Institute of the Tel Aviv Medical Center, which operates a specialized FMS clinic. Consecutive patients attending the clinic were offered to take part in the study, which was conducted between February 2013 and September 2015. Patients were recruited if a diagnosis of FMS had previously been confirmed by one of the study physicians, all of whom were experienced in the diagnosis. Both the ACR 1990 classification criteria [24] and the 2010-2011 modified preliminary ACR diagnostic criteria [2, 25] were acceptable for inclusion. Patients suffering from “secondary FMS” (i.e., FMS together with inflammatory joint disease) were excluded. Patients over the age of 18 were recruited.

RA was diagnosed by the participating physicians according to the ACR classification criteria [26]. RA patients with a clinical diagnosis of comorbid FMS were excluded. The study was approved by the institutional ethics review board (Helsinki committee) and all patients gave written informed consent prior to participation. No financial compensation was provided for participation. Participants were recruited consecutively during follow-up at the clinic and were handed the questionnaires with a standardized letter explaining the study and providing directions on proper completion of the questionnaires. Patients filled out the questionnaires on their own on-site and were offered assistance as needed by one of the researchers.

### 2.2. Measures and Questionnaires

Demographic data, including age, sex, marital status, educational level, and current professional status, were assessed by a questionnaire.

*FMS Severity*. The two components of the “polysymptomatic distress scale,” which are the components of the 2011 FMS diagnostic criteria, include the Widespread Pain Index (WPI) and the Symptom Severity Score (SSS). These scores were used in order to assess the severity of FMS symptoms among participating FMS patients.

### 2.3. Psychological Distress, Potential Anxiety, and Potential Depression

Similar to previous studies [23], we utilized the 4-item Patient Health Questionnaire-4 (PHQ-4) in order to determine mental health and illness [27, 28]. The PHQ-4 measures two of the DSM-IV criteria for major depression over a four-point scale, ranging from “0,” “not at all,” to “3,” “nearly every day.” Two other PHQ-4 items measure two DSM-IV criteria for general anxiety disorder while the total PHQ-4 score, which has a range between 0 and 12, is a measure of psychological distress [27].

### 2.4. Pain Related Disability

The pain disability index (PDI) rates the extent to which pain interferes with function across 7 areas: family/home responsibilities, recreation, social activity, occupation, sexual behavior, self-care, and life-support activity [29]. Each domain is rated using an 11-point scale ranging from 0 (no disability) to 10 (total disability). The total score ranges from 0 to 70.

### 2.5. Childhood Maltreatment

The 28-item short form version of the standardized Childhood Trauma Questionnaire (CTQ) is a highly reliable, well-validated self-report instrument that measures severity of different types of childhood and adolescence maltreatment (emotional, physical, and sexual abuse and emotional and physical neglect). The scores of each subscale range between 5 (no abuse or neglect) and 25 (maximal abuse or neglect). In addition, the instrument allows assessing elements of minimization/denial of maltreatment by 3 items with a score ranging from 0 to 3. Validated CTQ cut-off scores were used to detect any type of maltreatment and to grade maltreatment severity [30, 31].

### 2.6. Evaluation for PTSD

The Posttraumatic Diagnostic Scale (PDS), a well-validated instrument, was used in order to screen for patients suffering from PTSD [32]. In this questionnaire the patient is asked about the occurrence of a list of potential traumatic events involving either a threat to the individual or to a significant other person. Patients were also asked to indicate the occurrence of other potential traumatic events which had not been previously specified. In case the patient indicated more than one potential traumatic event, he or she was asked to state the most burdensome event and the year of its occurrence. In the second part of the questionnaire, the patients were asked about symptoms related to the aforementioned traumatic events. Thus, they were questioned about the presence of a symptom fulfilling the Diagnostic and Statistical Manual of Mental Disorders (DSM-IV) A2 criteria (intensive fear, shock, and helplessness) of PTSD [33]. In cases who reported a positive response on the A2 criterion, PTSD evaluation was performed by utilizing part 3 of the Posttraumatic Diagnostic Scale (PDS), based on the DSM-IV PTSD criteria.

This part of the PDS consists of 17 items which assess the three symptom clusters of PTSD (intrusions, avoidance, and arousal). The answers refer to the occurrence in the last month on a 4-point scale ranging from 0 (“not at all”) to 3 (“several times per week/almost always”). The items cover the criteria B (intrusive reexperiencing of the traumatic event in the form of flashbacks and nightmares, with an exaggerated response to trauma-related reminders/cues), C (persistent avoidance of trauma associated stimuli with emotional numbing), and D (persistent symptoms of exaggerated startle, increased physiological arousal, and sustained preparedness for an instant alarm response) according to DSM-IV. In addition, the duration of symptoms (criterion E: at least one month) and disability compared to the time before the traumatic event (criterion F) were assessed. PTSD diagnosis according to DSM-IV-TR criteria was determined by the PDS algorithm, which requires the following conditions: A1, A2, E, and F criteria being met and at least one B, at least three C, and at least two D criteria with scores ≥1. The sensitivity and specificity of the PDS compared with the DSM-IV structured clinical interview have been previously documented [34].

Severity of RA symptoms was assessed using the RA Disease Activity Index (RADAI). This is a highly reliable and validated self-administered measure of disease activity for clinical and epidemiological research regarding RA [35].

### 2.7. Statistical Analysis

Demographic and clinical parameters of the studied population were summarized: by mean ± standard deviation for continuous data and 95% confident interval for mean. Categorical descriptive data were presented as absolute values with percentages. Differences between groups were evaluated for continuous data by the Mann–Whitney *U* test and for categorical data by chi-square test. The association between probable PTSD based on retrospective reports on lifetime traumatic events and group (RA/FMS) was done by logistic regression model adjusted for PHQ. A *p* value of ≤0.05 was assumed to be significant.

Data were analyzed by SPPS Version 20.0.

## 3. Results

75 FMS and 23 RA patients were approached, all of whom agreed to participate in the study protocol. All participants were Israelis and all were Caucasian. As demonstrated in Table 1, over 97% of participants were Israelis of Jewish ethnicity while only 2.7% were Arabs. Various ethnic backgrounds common among the Israeli Jewish population (e.g., Ashkenazi Jews, Sephardic Jews, and mixed ethnicity) were represented in the study cohort. Educational level was significantly higher among FMS than among RA patients, with 71% reporting high school or university level education, compared with 22% among RA patients. These results were more similar to those previously reported among FMS patients in a US cohort (83.8% university education) when compared with a German cohort (16.9%). RA patients were significantly more likely to be married (Table 1). No significant differences were observed between FMS and RA patients regarding level of employment.

Table 2 presents differences in clinical parameters between the two study cohorts: FMS and RA patients. As shown in the table, RA patients suffered a significantly longer disease course. On the other hand, FMS patients demonstrated significantly higher scores on scales of mean pain disability, psychological distress, and Patient Health Questionnaire.

The severity of FMS symptoms, as expressed by the mean WPI and SSS, is presented in Table 3(a). Notably, the mean WPI among the Israeli FMS cohort was similar to that observed among American FMS patients (11.8 versus 11.2), which was higher than that observed among German FMS patients (7.6).

Table 3(b) presents clinical data regarding the level of disease activity of RA patients participating in the study, as assessed by the RADAI score.

Table 4 presents the results of the comparison of psychological distress, as assessed by the PHQ score, between Israeli FMS and Israeli RA patients. As noted from the results, FMS patients ranked significantly higher compared with RA patients on all components of the PHQ questionnaire, indicating significantly higher levels of psychological distress, anxiety, and depression.

Table 5 presents the results of probable posttraumatic stress disorder based on retrospective reports on lifetime traumatic events (assessed by the trauma list of PDS). As noted from the table, FMS patients exhibited a significantly higher prevalence of probable PTSD (37.3% versus 8.7%, resp., *p* = 0.009), with an OR of 6.26.

Subsequently, we performed a logistic regression in order to evaluate the OR of PTSD among FMS patients, when controlling for the PHQ components (i.e., controlling for potential anxiety and potential depression). The calculated OR was 4.9, with 95% CI 1.02–23.6 (*p* < 0.05).

We subsequently evaluated the frequency of retrospective report of childhood maltreatment of Israeli FMS patients compared with RA patients. The results of this evaluation, which is based on the results of the CTQ, are presented in Table 6. As noted from the results, FMS patients reported significantly higher levels of emotional abuse, emotional neglect, and physical neglect, when compared with RA patients. Interestingly, no significant difference was reported regarding either sexual abuse or physical abuse in general.

Notably, RA patients exhibited significantly higher levels of denial when compared with FMS patients.

We subsequently compared frequencies of “severe” and “very severe” CTQ scores between FMS and RA patients. As shown in Table 7, no significant difference could be demonstrated between the patient groups on any of these comparisons.

Finally, we compared our patient groups on the basis of health status reports, as assessed by the Short Form 36 (SF-36) health survey, as presented in Table 8.

As demonstrated in the table, FMS patients reported significantly lower scores on scales of energy/fatigue, emotional wellbeing, and social functioning, compared with RA patients, while no significant difference was observed on the scale of physical functioning.

## 4. Discussion

In the current study we have evaluated the role of childhood trauma in patients suffering from FMS, while comparing these patients to a control group of individuals suffering from chronic rheumatic disease, RA. This endeavor comes in continuation to previous research which has studied the cross cultural nature of the presumed relationship between childhood adversity and the development of chronic pain in general [36] and FMS in particular [23]. One of the most striking results observed in the current study was the high prevalence of probable PTSD (37.3%) among FMS patients, which was significantly higher than that observed among RA patients (8.7%). While the comorbidity of FMS and PTSD has been previously reported [12, 37, 38], to our knowledge, this is the first study to compare this issue between FMS and another classic chronic rheumatological disorder, RA. As psychiatric comorbidities such as depression and anxiety are important associates of many chronic illnesses [39], and particularly of those associated with chronic pain [40], it is particularly pertinent to evaluate the possible role played by childhood adversity in the unique case of FMS, as opposed to other conditions. Obviously, the high prevalence of PTSD in our cohort should not be interpreted as representing solely the consequences of childhood trauma; events occurring later in life would be expected to contribute to the occurrence of PTSD as well, although individuals experiencing childhood trauma may be considered more prone to develop PTSD in later life in the face of adversity.

As presented in our results, specific aspects of childhood maltreatment, such as neglect and emotional abuse, were particularly frequently reported by our cohort of FMS patients when compared with RA patients. Sexual and physical abuse, however, were not found to be significantly more frequent among our FMS patients. The possible association between sexual abuse during childhood and the adult development of FMS has been an issue of study and debate for over two decades [41, 42]. Nonetheless, despite the relative abundance of reports on this issue, a meta-analysis performed by Häuser et al. concluded that while the association could be confirmed, the level of evidence was poor [22]. In the transcultural study comparing German and American FMS populations [23], 22.5% of patients in both groups reported “severe or very sever sexual abuse” during childhood, compared with 9.3% of FMS patients in the current study. Thus, the lack of association between FMS and sexual abuse in the current study may be interpreted in various ways. Bona fide transcultural differences may exist between the Israeli population, which carries rather unique cultural, ethnic, and religious characteristics [43], in comparison with other populations such as Americans and Germans which have previously been studied in this aspect. Moreover, our results stress the importance attributable to less violent forms of childhood adversity, such as neglect, in the subsequent development of FMS. This is a finding worthy of further investigation. While acute and violent events, such as sexual victimization and physical abuse, might be assumed to cause massive activation of stress responses, including activation of the sympathetic nervous system, with possible long-term effects on future development of chronic pain, neglect would appear to be a more subtle type of trauma, for which other pathogenetic mechanisms may be more relevant. Thus, for example, maternal neglect during childhood has been reported to be associated with alterations in oxytocin and vasopressin signaling and the biology of attachment [43, 44]. This distinction between physical abuse and neglect has not been extensively addressed in the literature regarding FMS, which generally has tended to view both aspects as part of the same continuum [45]. The results are however in line with previous results reported by Van Houdenhove et al. regarding levels of victimization among patients suffering from chronic fatigue and fibromyalgia in a tertiary clinic [46].

In the current study, we have chosen to compare FMS patients with a control group of patients suffering from RA, which is a pathogenetically different type of chronic musculoskeletal disorder. While comparing FMS patients to healthy normal controls might have been an alternative approach, we considered RA patients, also suffering from chronic rheumatic disease, to be a more relevant group of reference. While traumatic childhood events have also been occasionally linked to the development of autoimmune disorders [47], the striking differences between the groups emphasize the role of childhood adversity in the etiology of FMS.

Walker et al. have performed a comparison of sexual, physical, and emotional abuse and neglect among FMS and RA patients nearly two decades ago [48]. Interestingly their results demonstrated that although childhood maltreatment was found to be a general risk factor for FMS, particular forms of maltreatment, such as sexual abuse per se, did not have specific effects, while physical assault in adulthood showed a strong and specific relationship with unexplained pain. These results compare with our results which failed to show a significant difference between FMS and RA patients regarding frequency of childhood physical and sexual abuse.

One interesting finding of the current study relates to the significantly higher rates of denial among RA patients when compared to FMS patients. While denial and similar “emotional oriented strategies” for coping with chronic illness have traditionally been considered to be avoidant and maladaptive tools for coping with stressful conditions such as chronic illness [49], recent research has demonstrated that, in the context of serious chronic illness such as renal failure, denial may actually have a positive effect in terms of coping and quality of life [50]. Thus, lower levels of denial among FMS patients may not invariably indicate resilience and case-by-case scrutiny must be implemented in order to evaluate whether denial is adaptive or maladaptive in each case.

An interesting and unexpected possible confounder of the current study arose as a result of the 2014 Israel-Gaza conflict (July-August 2014) which occurred during the recruitment of the study patients.

During this conflict, the Israeli population was affected by the recurrent firing of missiles from the Gaza strip into Israel, including Tel Aviv, where the study took place. As we and others have previously reported [51], civilian population exposed to this type of stress exhibits increased rates of a variety of somatic symptoms reminiscent of the FMS spectrum, as well as increased rates of anxiety. The effect of this type of stress on the recall of childhood adversities in the current study is difficult to quantify.

Additional limitations of the current study, as in similar previous attempts to study long-term effects of childhood adversity, result from the inherent recall and response bias associated with self-reported documentation of childhood trauma [36, 52]. This inherent limitation is difficult to circumvent, except through the meticulous documentation of childhood events based on external report (e.g., criminal documentation) which is also rarely exhaustive or comprehensive. Prospective follow-up of childhood trauma is one possible approach [53].

One final limitation towards generalization of our results is based on the fact that all FMS participants were recruited at a tertiary clinic specializing in the treatment of FMS. Patients reaching such a clinic may tend to be more complex and more refractory to treatment than patients encountered in primary care. Additional research into the role of childhood adversity in primary care patients, who may also be more likely to respond to specific therapeutic interventions, is called for.

## 5. Conclusion

In the current study we have demonstrated significantly higher rates of PTSD as well as childhood emotional abuse and physical and emotional neglect, among Israeli FMS patients, compared with RA patients, in the context of a tertiary healthcare facility. The FMS patient results were similar to those found in previous studies conducted in Germany and the USA, emphasizing the transcultural validity of the association between childhood adversity and FMS. Physical and sexual abuse were not found to be more frequently reported by FMS patients compared with RA patients, stressing the importance of less violent aspects of childhood and adversity such as neglect in the etiology of chronic pain.

## Figures and Tables

**Table 1:** Comparison of demographic data of Israeli patients with FMS (*n* = 75) and RA (*n* = 23).

	FMS	RA	*p* value
	*n* = 75	*n* = 23	
Mean age (mean, SD)	46.6 (12.7)	59.1 (12.0)	*p* < 0.001

Female gender *n* (%)	65 (86.7)	20 (87)	*p* = 0.971

Ethnicity			
Ashkenazi Jews *n* (%)	20 (26.7%)	7 (30.4%)	NS
Sephardic Jews *n* (%)	27 (36%)	13 (56.5%)	
Mixed *n* (%)	26 (34.7%)	3 (13.0%)	
Arabs *n* (%)	2 (2.7%)	0	

Marital status			
Single *n* (%)	26 (35.1)	1 (4.3)	*p* = 0.011
Married *n* (%)	31 (41.9)	16 (69.6)	
Divorced (%)	14 (18.9)	3 (13)	
Widowed (%)	2 (2.7)	3 (13)	
Other (%)	1 (1.4)	0	

Highest educational level			
Any educational level *n* (%)	1 (1.3)	0	*p* = 0.05
Primary school *n* (%)	3 (4)	1 (4.3)	
High school *n* (%)	28 (37.3)	16 (69.6)	
University *n* (%)	43 (57.3)	6 (26.1)	

Current professional status			
Without job *n* (%)	17 (22.7%)	3 (13.0%)	*p* = 0.4
Homemaker *n* (%)	8 (10.7%)	5 (21.7%)	*p* = 0.2
Part time job *n* (%)	22 (29.3%)	3 (13.0%)	*p* = 0.2
Full time job *n* (%)	23 (30.7%)	5 (21.7%)	*p* = 0.6
Pension *n* (%)	5 (6.7%)	6 (26.1%)	*p* = 0.02
Other *n* (%)	0	1 (4.3%)	*p* = 0.2

**Table 2:** Comparison of clinical data of Israeli FMS patients and RA patients.

	FMS *n* = 75	RA *n* = 23	*p* value
Mean years since diagnosis (mean, SD)	7.53 (5.6)	13.83 (10.4)	*p* = 0.01
Mean pain disability index (mean, SD) (0–70)	43.1 (16.1)	28.4 (19.8)	*p* = 0.003
Psychological distress Patient Health Questionnaire-4 total score (mean, SD) (0–12)	7.0 (4.7)	3.19 (3.8)	*p* = 0.005

**(a):** 

	FMS
	*n* = 75
Mean widespread pain index (mean, SD) (0–19)	11.8 (3.9)
Mean symptom severity score (mean, SD) (0–12)	8.85 (2.1)

**(b):** 

RADAI item	RA
	*n* = 23
Numerical rating scale questions (0–10)	
In general, how active has your arthritis been over the past 6 months? (Mean, SD)	6.2 (2.5)
In terms of joint tenderness and swelling, how active is your arthritis today? (Mean, SD)	5.3 (2.8)
How much arthritis pain do you feel today? (Mean, SD)	5.5 (3.2)

Likert scale question (0–6)	
Were your joints stiff when you woke up today? If yes, how long did this extra stiffness last? (Mean, SD) No = 0; <30 minutes = 1; 30 minutes to 1 hour = 2; 1-2 hours = 3; 2–4 hours = 4; >4 hours = 5; stiffness all day = 6	0.9 (1.1)

Joint list question (sum score range 0–24; 8 joints or joint groups, each graded 0–3)	
Please indicate the amount of pain you are having today in the joint areas listed below. (Mean, SD)^*∗*^ None = 0; mild = 1; moderate = 2; severe = 3 Shoulders, elbows, wrists, fingers, hips, knees, ankles, and toes	9.8 (6.0)

**Table 4:** Comparison of psychological distress of Patient Health Questionnaire score for Israeli FMS patients and RA patients.

	FMS	RA	*p* value
	*n* = 75	*n* = 23	
Psychological distress Patient Health Questionnaire 4 total score (mean, SD) (0–12)	7.0 (4.7)	3.2 (3.8)	*p* = 0.005

Psychological distress Patient Health Questionnaire depression disorder (mean, SD) (0–6)	3.1 (1.9)	1.9 (2.0)	*p* = 0.05

Psychological distress Patient Health Questionnaire anxiety disorder (mean, SD) (0–6)	3.8 (1.6)	2.8 (2.3)	*p* = 0.009

Patient Health Questionnaire potential depression disorder *n* (%)	44 (58.7%)	6 (26.1%)	*p* = 0.008 OR (95% CI): 4.0 (1.4–11.3)

Patient Health Questionnaire potential anxiety disorder *n* (%)	59 (78.7%)	12 (52.2%)	*p* = 0.02 OR (95% CI): 3.4 (1.3–9.1)

**Table 5:** Comparison of probable posttraumatic stress disorder based on retrospective reports on lifetime traumatic events (assessed by the trauma list of the PDS: frequency ≥10 in at least one group and DSM-IV diagnosis criteria for PTSD).

	FMS *n* = 75	RA *n* = 23	*p* value
Probable posttraumatic stress disorder *n* (%)	28 (37.3%)	2 (8.7%)	*p* = 0.009 OR (95% CI): 6.3 (1.4–28.7)

**Table 6:** Comparisons of retrospective reports of childhood maltreatment of Israeli patients with FMS and RA.

	FMS *n* = 75	RA *n* = 23	*p* value
Emotional abuse CTQ (5–25) (mean, SD)	9.4 (5.0)	7.0 (4.8)	*p* = 0.003
Physical abuse CTQ (5–25) (mean, SD)	6.9 (4.0)	5.9 (2.7)	*p* = 0.3
Sexual abuse CTQ (5–25) (mean, SD)	7.38 (4.16)	6.2 (3.1)	*p* = 0.1
Emotional neglect CTQ (5–25) (mean, SD)	10.81 (5.61)	7.8 (4.1)	*p* = 0.01
Physical neglect CTQ (5–25) (mean, SD)	7.72 (3.1)	6.0 (1.9)	*p* = 0.04
Denial *n* (%)	25 (33.3%)	17 (73.95%)	*p* = 0.0008

**Table 7:** Comparison of retrospective reports on childhood adversities (assessed by Childhood Trauma Questionnaire) of Israeli FMS patients and RA patients.

	FMS *n* = 75	RA *n* = 23	*p* value
Severe and very severe emotional abuse CTQ (16–25) *n* (%)	9 (12.0)	2 (8.7)	*p* = 1.000 OR (95% CI): 1.4 (0.3–7.2)

Severe and very severe physical abuse CTQ (13–25) *n* (%)	7 (9.3)	1 (4.3)	*p* = 0.676 OR (95% CI): 2.3 (0.3–19.4)

Severe and very severe sexual abuse CTQ (13–25) *n* (%)	7 (9.3)	2 (8.7)	*p* = 1.000 OR (95% CI): 1.1 (0.2–5.6)

Severe and very severe emotional neglect CTQ (18–25) *n* (%)	12 (16.0)	1 (4.3)	*p* = 0.3 OR (95% CI): 4.2 (0.5–34.1)

Severe and very severe physical neglect CTQ (13–25) *n* (%)	6 (8.0)	0 (0)	*p* = 0.331 OR (95% CI): 1.1 (1.0–1.2)

**Table 8:** Comparison of health status reports (assessed by the Short Form (36) health survey) of patients with FMS and patients with RA (*t*-test).

	FMS *n* = 75	RA *n* = 23	*p* value
Physical functioning (mean, SD) (0–100)	45.07 (21.5)	51.1 (21.2)	*p* = 0.2
Energy/fatigue (mean, SD) (0–100)	29.8 (12.72)	48.0 (15.5)	*p* < 0.001
Emotional wellbeing (mean, SD) (0–100)	42.2 (16.3)	59.8 (18.8)	*p* < 0.001
Social functioning (mean, SD) (0–100)	39.5 (27.1)	62.0 (35.2)	*p* = 0.002
